# Immunogenetic and tolerance strategies against a novel parasitoid of wild field crickets

**DOI:** 10.1002/ece3.6930

**Published:** 2020-10-26

**Authors:** Kristin L. Sikkink, Nathan W. Bailey, Marlene Zuk, Susan L. Balenger

**Affiliations:** ^1^ Department of Biology University of Mississippi Oxford MS USA; ^2^ Centre for Biological Diversity School of Biology University of St Andrews St Andrews UK; ^3^ Department of Ecology, Evolution, and Behavior University of Minnesota‐Twin Cities St. Paul MN USA

**Keywords:** *Ormia ochracea*, resistance, *Teleogryllus oceanicus*, transcriptomics

## Abstract

Among the parasites of insects, endoparasitoids impose a costly challenge to host defenses because they use their host’s body for the development and maturation of their eggs or larvae, and ultimately kill the host. Tachinid flies are highly specialized acoustically orienting parasitoids, with first instar mobile larvae that burrow into the host’s body to feed. We investigated the possibility that *Teleogryllus oceanicus* field crickets employ postinfestation strategies to maximize survival when infested with the larvae of the parasitoid fly *Ormia ochracea*. Using crickets from the Hawaiian Islands of Kauai, where the parasitoid is present, and crickets from the Cook Islands (Mangaia), where the parasitoid is absent, we evaluated fitness consequences of infestation by comparing feeding behavior, reproductive capacity, and survival of males experimentally infested with *O. ochracea* larvae. We also evaluated mechanisms underlying host responses by comparing gene expression in crickets infested with fly larvae for different lengths of time with that of uninfested control crickets. We observed weak population differences in fitness (spermatophore production) and survival (total survival time postinfestation). These responses generally did not show an interaction between population and the number of larva hosts carried or by host body condition. Gene expression patterns also revealed population differences in response to infestation, but we did not find evidence for consistent differences in genes associated with immunity or stress response. One possibility is that any postinfestation evolved resistance does not involve genes associated with these particular functional categories. More likely, these results suggest that coevolution with the fly does not strongly select for either postinfestation resistance or tolerance of parasitoid larvae in male crickets.

## Graphical abstract

Experimental infestation of field crickets with *Ormia ochracea* larvae strongly impacted gene expression profiles of their hosts, and these varied over the time course of infestation. Coevolving crickets from Kauai showed a larger magnitude and extent of differential gene expression in response to infestation than did crickets from a non‐coevolving population (Mangaia). Host immune and stress response genes, however, responded similarly to infestation with *O. ochracea* larvae, regardless of population coevolutionary history. We found little evidence for population‐level differences in immunity, tolerance, or fitness measures in response to infestation, suggesting that evolved pre‐infestation avoidance measures are under stronger selection in the Kauai population.

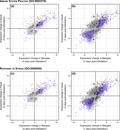

## 1 INTRODUCTION

Different types of host–parasite relationships are characterized by highly variable costs to the survival and reproduction of both partners in the interaction. To understand the evolution of such host–parasite arms races, it is useful to evaluate these costs and to identify the underlying mechanisms of host responses (Howick & Lazzaro, 2017; Lenz et al., 2012; Medina & Langmore, 2016). Extreme examples involving highly virulent parasites can be particularly instructive in elucidating selection for host resistance and tolerance strategies. Parasitoids impose a particularly costly challenge to their host, because the development and maturation of their eggs and/or larvae results in the death of their insect host (Godfray, 1994). Endoparasitoids live within the body of their host, obtaining shelter and nutrition during development. When endoparasitoids are introduced as eggs, hosts can resist further development and maturation into larval stages by early detection followed by encapsulation of eggs, which causes parasitoid asphyxiation and triggers production of cytotoxic substances (Kraaijeveld & Godfray, 1999, 2009; Lavine & Strand, 2002). However, some parasitoids, such as tachinid flies, release mobile first instar larvae, or planidia, on and around potential hosts (Adamo et al., 1995; Cade, 1975). Larvae actively burrow into the host’s body. They remain mobile until emergence, which allows them to avoid complete encapsulation and its fatal consequences due to asphyxiation (Stireman et al., 2006). The ability of such parasitoids to thwart common host defenses thus poses a unique set of challenges for their hosts. It also represents a powerful opportunity to characterize genetic pathways that respond to an extreme selection pressure (Zuk et al., 1993), and to test the evolutionary limits of different host strategies when parasites appear to be “winning” the arms race, by comparing the ability of hosts from populations with different coevolutionary histories to tolerate and resist infestation (Råberg et al., 2009).

Host resistance and tolerance to infection are not mutually exclusive; resistance is typically evaluated with respect to infection intensity, while tolerance is determined by the slope of the regression of host fitness relative to infection intensity (Råberg et al., 2009). Individuals with relatively greater resistance have lower absolute parasite loads due to host behavioral avoidance or immune activity after infection (Boots, 2008). In contrast, tolerance does not affect the fitness of the parasite and is instead measured as the relative decline in host health or fitness across varying levels of parasite burden (Howick & Lazzaro, 2017; Råberg et al., 2009). Functionally, more tolerant hosts do not control infection load, but instead invest in adaptive tissue damage control (Medzhitov et al., 2012; Råberg et al., 2009; Soares et al., 2014). For example, when parasitoid infestation occurs in reproductively mature adult hosts, investment in immunity may be costly and require decreased investment in reproductive opportunities. Adamo et al. (1995) found that field crickets infested with tachinid fly larvae exhibited reduced mating and competitive behaviors, suggesting decreased reproductive success postinfestation, which may be due at least in part to an upregulation of immune activity. In insects, costs of an immune response can arise from the production of attack cells and melanin (Siva‐Jothy et al., 2005). Although also harmful to the host, cytotoxic products generated during the formation of melanin (e.g., ROS and quinones) cause general cell damage to parasitoids (Siva‐Jothy et al., 2005). It is possible that, even in the absence of complete encapsulation, an increased melanization response that generates high concentrations of such toxins could retard larval growth and development, thus still allowing for increased host longevity and reproduction due to increased immune activation. This study examines the limits and trade‐offs of these strategies, and whether adaptive responses are likely to evolve in response to the introduction of a novel parasitoid.

We investigated cricket host responses to infestation by Tachinid flies of the tribe Ormiini. These parasitoids are highly specialized; they all attack crickets and katydids (Ensifera, Orthoptera) and locate their preferred host by eavesdropping on the male hosts’ acoustic sexual signals (Allen, 1995; Cade, 1975; Lehman, 2003). Perhaps the most well‐studied species is *Ormia ochracea*, which parasitizes many field crickets in the southern United States and northern Mexico. In this study, we took advantage of the fly’s recent range expansion in the Hawaiian archipelago, where *O. ochracea* was likely incidentally introduced sometime between the arrival of the first traders from the continental United States in the late 18th century and its discovery in 1991 (Gray et al., 2019; Zuk et al., 1993). *O. ochracea* has become established on at least three islands where it parasitizes the field cricket *Teleogryllus oceanicus*, which has also been introduced to Hawaii (Tinghitella et al., 2011; Zuk et al., 1993). These two species are only known to co‐occur on the Hawaiian Islands, and this evolutionarily recent host–parasitoid relationship has led to changes in male crickets’ song characteristics and calling activity (Kolluru, 1999; Zuk & Kolluru, 1998). The flies have also driven the evolutionary loss of male song through multiple mechanisms. Populations on at least three Hawaiian Islands, including the one used in the current study, contain males with novel wing mutations ("flatwing") that extinguish male acoustic signaling and protect them from the fly (Zuk et al., 2006).

While multiple behavioral strategies are employed by *T. oceanicus* and other species of field crickets to avoid infestation by *O. ochracea* (Lewkiewicz & Zuk, 2004; Vincent & Bertram, 2010b; Zuk et al., 1993), and morphological adaptations have evolved in response to the flies (Pascoal et al., 2016; Rayner et al., 2019; Zuk et al., 2006), neither of these are any help once the larva(e) become established in a cricket host. Males capable of singing, and thus attracting the fly, persist in Hawaii (Rayner et al., 2019; Zuk et al., 2018). Additionally, some silent males have been observed to harbor *O. ochracea* larvae in the wild [Bailey and Pascoal, personal Observation]; thus, selection may also be operating on resistance and tolerance of *T. oceanicus* following infestation.

Here, we ask whether postinfestation strategies might be employed by *T. oceanicus* crickets to maximize survival when infested with *O. ochracea*. Such strategies could involve resistance or tolerance or both, although we hypothesize that tolerance would be more effective at increasing survival and reproduction due to the highly mobile nature of the parasitoid larvae once in the host. Thus, we predict that crickets from a coevolving population would show responses to infestation consistent with a tolerant phenotype. Using crickets from the Hawaiian Islands of Kauai, where the parasitoid co‐occurs, and crickets from the Cook Islands (Mangaia), where the parasitoid is absent, we first compared host body mass across the infestation, reproductive capacity (spermatophore production and retention of testes), and survival of males following infestation with *O. ochracea* larvae. If increased host mass benefits not only the parasitoid (Adamo, et al., 1995; Beckers & Wagner, 2011) but also the host, then we expect Kauai crickets to gain more mass relative to Mangaia crickets, and that such mass gain will lead to an increased likelihood of retaining reproductive capabilities and overall length of survival. We then evaluated molecular mechanisms underlying host responses by comparing gene expression in crickets infested with fly larvae for different lengths of time against that of uninfested control crickets. Population differences in the transcriptomic response to infestation were of particular interest. If Kauai hosts are under positive selection for resistance to this parasitoid, then infestation should provoke a stronger gene expression response: When they are infested, we expect this population to show greater levels of differential expression of genes associated with immunity relative uninfested controls than would be observed in infested versus control crickets from Mangaia. If, as we hypothesize, Kauai hosts are under positive selection for tolerance to the fly, then we expect this population to differ overall in levels of expression of genes relative to Mangaia crickets, irrespective of the genes involved. Although recent studies of *Drosophila melanogaster* have suggested that secretory and metabolic pathways show altered regulation in hosts with greater bacterial pathogen tolerance (Dionne et al., 2006; Howick & Lazzaro, 2017; Lissner & Schneider, 2018; Troha et al., 2018), it remains unclear what if any specific functional categories of genes are associated with increased tolerance to parasitoids. We therefore do not make predictions regarding differential expression of any specific functional group of genes in relation to tolerance, focusing instead on overall dissimilarity of transcriptional responses and coexpressed gene modules between populations.

## 2 MATERIALS AND METHODS

### 2.1 Experimental design

#### 2.1.1 Cricket populations and rearing

Colonies of crickets derived from two island populations were established and maintained in the laboratory. All crickets were reared in the absence of parasitoid flies. The Hawaiian crickets originated from the island of Kauai (22°04'N −159°29'W); the Kauai colony was first established in 2003 and is supplemented with the offspring of 10 to 20 wild‐caught females approximately annually (Bailey et al., 2010). Regular Kauai colony supplementation ensures that genes under selection in the wild by the parasitoid are continually reintroduced to the laboratory population. The Mangaia Island (−21°55'S −157°55'W) colony was similarly established in the laboratory in 2009 from the offspring of approximately 20 adult females but was not supplemented again before the start of this study in 2013 (Balenger & Zuk, 2015; Pascoal et al., 2016). To minimize the possibility that laboratory stock from the different islands experienced different founder or bottleneck effects, we maintained both at a minimum of 100 breeding adults at all times. Infestations described in this study were conducted in 2013–2014. Colonies were housed in groups of 25–40 adults in 15‐L containers in temperature‐, humidity‐, and light‐controlled incubators set at 26°C and 75% humidity with a 12:12 photoreversed light:dark schedule. Juvenile males were removed from their colony prior to their penultimate molt when sex differences became apparent, but wing morph was not yet distinguishable. They were transferred to individual 118‐mL containers containing Teklad high‐fiber rabbit chow, a small piece of water‐soaked cotton, and egg carton material for shelter. Individuals remained under these conditions for the duration of the experiment.

#### 2.1.2 Fly collection

Collecting and transporting gravid flies from Hawaii was prohibitively difficult. Mitochondrial haplotypes and microsatellite genotypes support a western US origin of populations of *O. ochracea* across the Hawaiian Islands (Gray et al., 2019 ). Flies were therefore captured in the Santa Monica Mountains, Los Angeles County, California. Synthetic songs of two local host species, *Gryllus integer* and *G. lineaticeps*, were broadcast from portable speakers placed inside of slit traps designed for acoustic trapping of *Ormia* (Paur & Gray, 2011; Walker, 1989). Only gravid female flies are attracted to these acoustic traps. All captured flies were transferred to plastic containers with twigs and paper towels soaked in water and shipped overnight to the University of Minnesota (UMN). Upon arrival at UMN, female flies were held for 1–2 days in mesh insect cages (12” × 12” × 12”) containing cotton soaked in a 10% organic honey water solution. Flies were maintained separately from crickets in an incubator at 26°C and 75% humidity with a 12:12 photoreversed light:dark schedule until infestation (Vincent & Bertram, 2010a).

#### 2.1.3 Infestation

Infestation protocols followed those of previous studies (Bailey & Zuk, 2008; Vincent & Bertram, 2010a). Sexually mature adult male crickets were artificially infested with *O. ochracea* planidia. Within 48 h of arrival at UMN, first instar planidia were dissected from gravid female flies. Each cricket was manually infested with two planidia by transferring them on the tip of a dissecting probe onto the body of the cricket under the junction between the thorax and the abdomen (Bailey & Zuk, 2008; Vincent & Bertram, 2010a). In the wild, typically only one or two larvae emerge from *T. oceanicus* (Zuk et al., 1993); therefore, we used two planidia to simulate a natural level of infestation. Crickets were then returned to the incubator in their individual containers and left undisturbed for 24 h. Crickets in the control group were handled in an identical manner and for the same amount of time, except that no larvae were transferred on the probe. All Mangaia crickets expressed a normal‐wing phenotype, while both normal‐wing and flatwing males from the Kauai population were included in the study. Although approximately 95% of males in the wild population on Kauai expressed a flatwing phenotype at the time, the laboratory colony generally has a 1:1 wing morph ratio. Kauai crickets were haphazardly assigned to treatment group with respect to wing morph; thus, each treatment group for this population includes approximately equal numbers of normal‐wing and flatwing males.

### 2.2 Host survival and fitness experiment

#### 2.2.1 Sampling and measurement of survival and fitness

To study the effects of infestation on host survival and fitness, 184 adult crickets between 5 and 6 days after adult eclosion were infested (*n* = 73 from Kauai; *n* = 111 from Mangaia), and 133 were uninfested controls (*n* = 52 from Kauai; *n* = 81 from Mangaia). Crickets were weighed to the nearest 0.001 g within one hour prior to infestation (Day 0). Pronotum length was measured twice to the nearest 0.01 mm, and the mean was used to quantify body size. Each individual was weighed daily between 15:00 and 17:00 until larval emergence occurred, or once 10 days had passed since infestation. Crickets were checked every 2–3 h for larval emergence. We removed the ampulla, the portion of the spermatophore containing sperm and protruding outside the body, from infested crickets by manually extracting it with forceps. This was done five days following infestation, and we noted whether a new spermatophore was present the following day (Day 6). Time to emergence of at least one larva was recorded and used to calculate host survival time, as infested crickets die following larval emergence. A window of 2–3 h was sufficient for such a calculation because, although fly larvae pupate within one hour of emergence (S. L. Balenger, personal observation), we removed all emerged *O. ochracea* larvae from containers prior to pupation. Wet weight was immediately collected from each larva, and the number of larvae to emerge from each cricket was recorded. The relationship between parasite load and host fitness is critical to evaluating tolerance; thus, crickets were returned to incubators and we continued to check for any further larvae until host death. Following death, crickets were stored at −20°C until they could be dissected. Crickets were dissected to determine whether testes were still present and whether there were any remaining larvae.

We removed 18 infested crickets from the survival and fitness analyses for the following reasons: (a) No larvae had emerged from crickets (Mangaia: *n* = 9 (8%); Kauai: *n* = 7 (9%)) ten days following infestation and no larvae were found in the host during dissection; and (b) a larva remained inside the host body after death (Kauai: *n* = 2). Sample sizes vary slightly between analyses of mass and body condition because pronotum width was not collected for 2 and 17 individuals from the Mangaia and Kauai populations, respectively.

#### 2.2.2 Statistical analyses

All statistical analyses were performed using SAS 9.4 (SAS Institute). Although measures of immune activity have not been found to significantly differ between the two wing morphs when exposed to standard rearing conditions (Bailey et al., 2011, Balenger et al. 2018), we first evaluated whether response to infestation with *O. ochracea* differed between Kauai males with respect to wing morph. Using repeated‐measures multivariate ANOVA (PROC MIXED) with a compound symmetry covariance structure, we examined the effect of wing morph on body condition over the course of the infestation. An index of body condition was calculated as the residuals from a regression of mass relative to pronotum length. We refer to these residuals as an index based on the recognition that larval mass contributes to overall mass of infested crickets; thus, larger residuals should not necessarily be interpreted as better host condition or health. Treatment group and number of days since infestation (0–6) and all interaction terms were also included in the model as fixed effects. We examined body condition up to the sixth day of infestation only because most infested hosts had at least one larva emerge before mass was collected on the seventh day. Overall significance of groups (wing morph, treatment, and days infested) was first evaluated using type III tests. We determined differences among groups at each level using least squares means with the slice option, or analysis of simple effects within interaction terms, for any term that was significant in the full model. We then used a Wald chi‐square test to examine whether wing morphs differed in the number of larvae to emerge. We also performed factorial logistic regressions (PROC LOGISTIC) to test for effects of wing morph and number of larvae to emerge and their interaction on whether (a) male crickets produced a spermatophore six days following infestation and (b) their testes were present upon dissection.

We also used repeated‐measures multivariate ANOVAS (PROC MIXED) with a compound symmetry covariance structure to specifically examine the effect of population of origin on body condition over the course of the infestation. Treatment group and days infested (0–6) and all interaction terms were also included in the model as fixed effects. Overall significance of groups (population, treatment, and days infested) was first evaluated using type III tests. We then determined differences among groups at each level using least squares means with the slice option for any term that was significant in the full model. We used a Wald chi‐square test to examine whether populations differed in the number of larvae to emerge and whether the presence of testes at the time of host death was related to spermatophore production six days postinfestation (late stage infestation). We constructed fully factorial logistic regressions using PROC LOGISTIC to evaluate whether population of origin, number of larvae to emerge, and host body condition preinfestation and six days following infestation predicted whether (a) male crickets produced a spermatophore six days following infestation and (b) their testes were present upon dissection. These models were assessed using joint Wald chi‐square tests.

A Cox proportional hazards regression survival analysis was also performed to evaluate whether host population of origin or number of larvae to emerge predicted host survival time postinfestation. To examine interaction effects, we further constructed fully factorial general linear models to evaluate the effects of the number of larvae to emerge within ten days following infestation, host population of origin, and host body condition preinfestation and six days following infestation in determining the total mass of larvae to emerge, the time to emergence of the first larva, and the total host survival time postinfestation. These models were assessed using type III sums of squares.

### 2.3 Gene expression study

#### 2.3.1 Sampling and tissue collection

To study the effects of infestation on gene expression, a separate group of 57 male crickets between 4 and 12 days after eclosion were infested (*n* = 38 from Kauai; *n* = 19 from Mangaia), and 20 were uninfested controls (*n* = 9 from Kauai; *n* = 11 from Mangaia). Among infested crickets, tissues were collected either four days following infestation (*n* = 18 from Kauai; *n* = 9 from Mangaia) or seven days following infestation (*n* = 20 from Kauai; *n* = 10 from Mangaia). These time points were chosen based on previous behavioral studies demonstrating that infested males do not differ in courtship or reproductive behaviors until four days after infestation, but are significantly different seven days after infestation (Adamo et al., 1995, but see Beckers & Wagner, 2011). Each individual in this study was weighed and measured prior to infestation similar to the description given above. All crickets were again weighed four days later. Control crickets and infested crickets in the seven‐day treatment group were weighed once more seven days following infestation. Between six and seven days after infestation, larvae naturally emerged from six Kauai crickets and three Mangaia crickets; these were therefore excluded from the study.

Cricket head and body tissues were separated and the digestive tract discarded. Samples were immediately stored in RNAlater Solution (Ambion) at 4°C for 24 h. The following day, samples were placed at −80°C until RNA extraction. Immediately prior to extraction, pronotum, wings, and legs were removed from the bodies; larvae were then dissected from the abdomen. We extracted RNA from cricket bodies using RNeasy Plus kits with gDNA eliminator spin columns (Qiagen) according to the manufacturer’s directions. Ultimately, six individuals from each treatment group (control, four days postinfestation, and seven days postinfestation) and population (Kauai and Mangaia) were included in the study. Because of the prohibitive cost of sequencing all individuals from all treatments, total RNA was pooled prior to library preparation such that each sample included equal amounts of RNA from two individuals of the same treatment group. The three pooled samples from Kauai each contained RNA from one flatwing individual and one normal‐wing individual, while the three pooled samples from Mangaia each contained RNA from two normal‐winged individuals.

#### 2.3.2 Library preparation, sequencing, and read processing

Sequencing libraries enriched for mRNA were prepared using the TruSeq RNA Library Preparation Kit v2 (Illumina) at the University of Minnesota Genomics Center (UMGC; Minneapolis, MN). The 18 pooled samples were multiplexed and sequenced in a single lane on an Illumina HiSeq 2000 at UMGC. In total, we generated 188.7 million paired‐end reads 50 nt in length.

After demultiplexing, reads were cleaned and trimmed using Trimmomatic (version 0.33) (Bolger et al., 2014). We removed all sequences that matched to Illumina TruSeq adapters. In addition, we trimmed sequence reads when the average quality score within a sliding window of 4 bp reached *Q* < 5. Finally, any reads shorter than 25 bp in length after trimming were discarded. Trimmed reads were aligned to the *T. oceanicus* genome assembly (Pascoal et al., 2019) using the STAR alignment software (version 2.5.3a) (Dobin et al., 2012). Because STAR cannot process datasets containing both single‐ and paired‐end reads, and because the majority of filtered reads contained both members of the pair, we elected to use only the sequences for which both reads of a pair passed quality filters in the alignments and subsequent analyses. For the first STAR mapping pass, reads were aligned using default parameters and a reference set of exon splice junctions that had been previously annotated in the reference genome (Pascoal et al., 2019). Following the first alignment, novel splice junctions inferred from all samples were merged using StringTie (version 1.3.4d) (Pertea et al., 2015. The number of reads aligned to each *T. oceanicus* gene was quantified following a second alignment to the genome with the novel splice junctions identified.

#### 2.3.3 Gene expression profiling

All statistical analyses on gene expression data were conducted using R (v. 3.5.2) (R Development Core Team, 2018). We first used the edgeR package (McCarthy et al., 2012; Robinson et al., 2009; Robinson & Oshlack, 2010) to normalize read counts and to test for differential expression using a negative binomial generalized log‐linear model. We used specific contrasts to test for differential expression between populations, time since infestation, or the interaction. We adjusted for multiple comparisons using the Benjamini–Hochberg method (Benjamini & Hochberg, 1995).

We used nonmetric multidimensional scaling (nMDS), an unsupervised ordination method, to visualize overall patterns of expression among the samples. We excluded from this analysis any genes that did not appear at a minimum threshold of 1 count per million reads (about 6–7 total reads, given our sequence coverage for each sample) in at least two samples. Read counts were normalized using the TMM implemented in edgeR (Robinson & Oshlack, 2010). We used a Bray–Curtis dissimilarity matrix (Bray & Curtis, 1957); using other distance or dissimilarity metrics did not substantially alter the results of the ordination. Data transformation, ordination, and scaling were performed in *k* = 4 dimensions using the vegan package (Oksanen et al., 2013). We also tested for significant differences between populations and infestation stage with a permutational analysis of variance on the Bray–Curtis dissimilarity matrix using the vegan package. Population, time since infestation, and the interaction term were included as factors in the model. 1,000 total permutations were run.

#### 2.3.4 Coexpression network analysis

We performed weighted gene coexpression network analysis using the R package WGCNA (Langfelder & Horvath, 2008). To improve computation time, we excluded the 40% of genes with the lowest variance across treatments (i.e., those genes least likely to be informative). Gene counts were normalized and transformed using the voom method implemented in limma (Law et al., 2014; Ritchie et al., 2015). The signed coexpression network was constructed using the WGCNA package function with the option for automatic blockwise module construction, with the maximum block size set to 10,000 genes and the following parameters: networkType = signed hybrid, deepSplit = 2, minModuleSize = 30, pamRespectsHybrid = FALSE, mergeCutHeight = 0.15.

We further used a bootstrapping approach to assess the stability of the modules identified by WGCNA and to identify coexpressed gene modules that were strongly supported in our data. Briefly, we used the function “sampledBlockwiseModules” in the WGCNA package to resample 18 samples from our original data with replacement and reconstruct the network topology with the same parameters. We created 250 bootstrapped networks in this way. For each gene in the original network, we assessed the reliability of its assignment to a module using the following approach: (a) If a module in a resampled network contained at least 10% of the genes in a module in the original network, then those overlapping genes from the resampled module were considered as corresponding to the original module. Note that the resampled modules could match to more than one original module, and genes from the resampled modules could be divided among the original module assignments based on this criterion. Thus, the final network topology will be biased toward the modules created from the full set of observed data. (b) To construct the consensus network topology, we considered that a gene was reliably part of the original assigned module if it was also assigned to that module in at least 70% of the bootstrap samples. Genes that did not meet both of the above criteria were removed from their modules and moved to the “Unassigned” group. Finally, we merged modules with highly correlated eigengene expression (*r* > .85). Remaining modules that contained fewer than the minimum module size of 30 genes were also moved to the “unassigned” bin.

The eigengene for each module, defined as the first principal component of the expression of all the genes in the module, was calculated to represent the general pattern of expression seen within each module. We performed an analysis of variance on eigengene expression for each module to test for effects of population, infestation time, and population‐by‐infestation interactions on the overall expression of the module genes.

#### 2.3.5 Gene annotation and enrichment analysis

For the merged set of all transcripts identified from the second‐pass STAR alignment, we used a translated BLAST query (blastx) (Altschul et al., 1990, 1997; Camacho et al., 2009) to identify homology to genes in the NCBI nr protein database. Transcripts were determined to have significant homology if E < 1E‐5. The significant blastx hits were used to map gene ontology (GO) terms (The Gene Ontology Consortium, 2000, 2015) to transcripts with the software package Blast2GO (version 3.3.5) (Conesa et al., 2005; Conesa & Götz, 2008; Gotz et al., 2008) using default parameters for annotating GO matches. To test for enrichment of GO annotation terms in coexpression modules, we used the R package topGO (version 2.34.0)(Alexa et al., 2006). We applied a Fisher exact test using the “classic” method to identify terms significantly enriched for each module. A total of 4983 genes with mapped GO annotations could be used in these analyses.

## 3 RESULTS

### 3.1 Survival and fitness—Kauai wing morphs

Among Kauai crickets, control and infested individuals included similar proportions of each wing morph (flatwing: control = 42%; infested = 48%). Repeated‐measures analyses of body condition identified a significant interaction effect between treatment and day (Table 1), such that infected crickets had significantly greater body condition than controls on Days 4, 5, and 6 (Table S1). Wing morphology was not identified as a significant predictor of body condition, either on its own or as part of any interaction terms (Table 1).

**TABLE 1:** Results of repeated‐measures ANOVA least squares effect test examining the effects of wing morphology (flatwing or normal), treatment (control or infested), and day of infestation (D0‐6) on host body condition. *p*‐values < 0.05 are bolded

	Body condition		
	df	*F*	*p*
Morph	1	2.34	.13
Treatment	1	2.83	.10
Day	1	1.73	.19
Morph x Treatment	6	0.95	.46
Morph x Day	6	0.38	.89
Treatment x Day	6	13.78	**<.001**
Morph x Treatment x Day	6	0.99	.43

The number of larvae to emerge also did not significantly differ between morphs, although the likelihood of 2 larvae emerging tended to be greater in flatwing males (*χ* ^2^ = 3.62, *p* = .06). Logistic models showed that the likelihood of producing a spermatophore or retaining testes until death was not significantly predicted by wing morph (spermatophore: *χ* ^2^ = 0.01, *p* = .95; testes: *χ* ^2^ = 0.08, *p* = .78), the number of larvae to emerge (spermatophore *χ* ^2^ = 1.87, *p* = .17; testes *χ* ^2^ = 0.34, *p* = .56), or their interaction (spermatophore *χ* ^2^ = 1.12, *p* = .29; testes *χ* ^2^ = 1.89, *p* = .17). In all, wing morph showed no significant effects on the variables of interest, and therefore was excluded as a factor in all subsequent analyses.

### 3.2 Survival and fitness—Mangaia and Kauai populations

**FIGURE 1:**
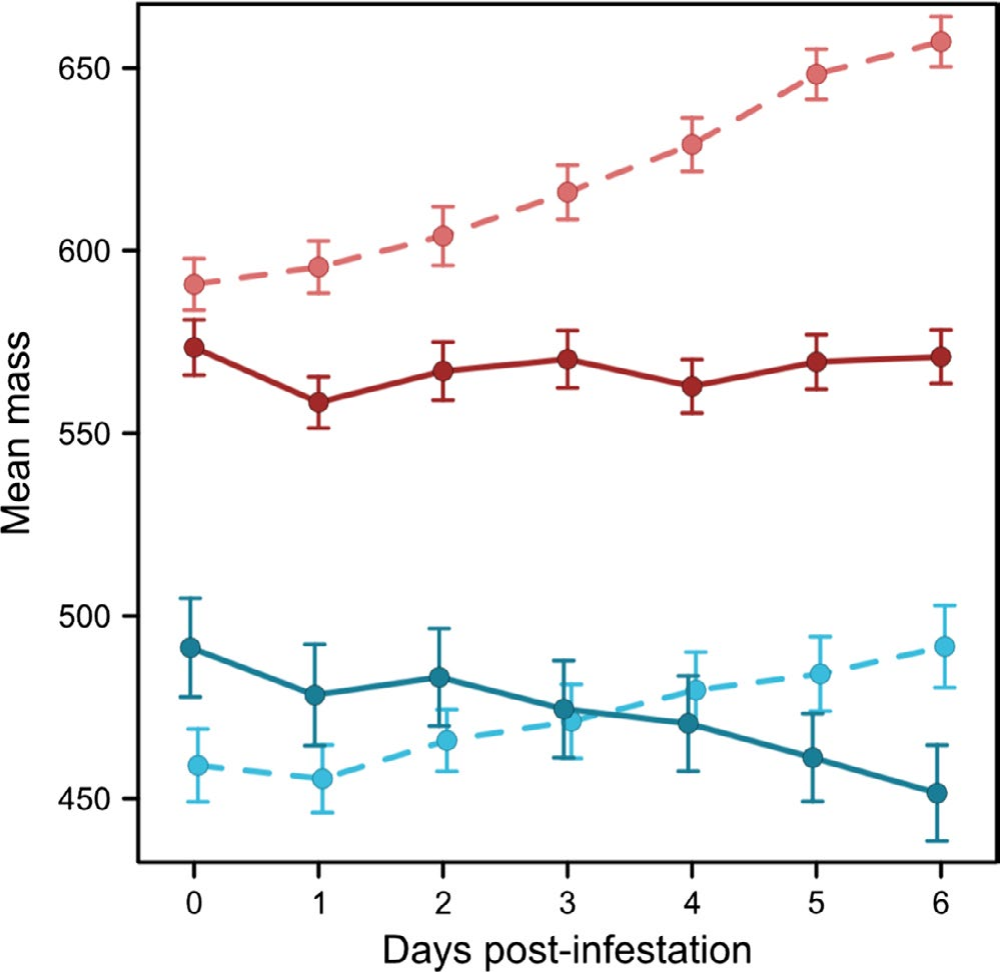
Male *Teleogryllus oceanicus* mass differs between crickets derived from Kauai and Mangaia populations. Infested males gain mass over the course of the infestation with *Ormia ochracea*. Shown are means with standard error bars for control (dark circles) and crickets infested with one or 2 larvae (light circles) from each population (red—Mangaia; blue—Kauai). Dashed lines connect mean control cricket mass over time; solid lines connect mean infested cricket mass over time

Preinfestation mass and mass six days postinfestation were significantly different between the two populations (Day 0 mass: *t* = 11.45, *p* < .001; Day 6 mass: *t* = 13.90, *p* < .001), such that crickets derived from the Mangaia population were heavier than those from Kauai (Figure 1). We therefore chose to use body condition in subsequent tests to control for interactions between population of origin and body mass.

Repeated‐measures analysis of body condition identified significant effects of treatment and interaction terms between treatment and population, and treatment and day (Table 2). Infected crickets had significantly higher body condition overall, such that, regardless of population of origin, infected crickets were larger than controls on Days 3, 4, 5, and 6 (Table S2). As predicted, we found a significant interaction between population and treatment, with crickets from Mangaia gaining relatively more mass over the course of the infestation than those from Kauai (Table 2, Figure 1).

**TABLE 2:** Results of repeated‐measures ANOVA least squares effect test examining the effects of population (Kauai or Mangaia), treatment (control or infested), and day of infestation (D0‐6) on host body condition. p‐values < 0.05 are bolded

	Body condition		
	df	*F*	*p*
Population	1	0.33	.57
Treatment	1	18.73	**<.001**
Day	1	0.20	.98
Population × Treatment	6	8.08	**<.005**
Population × Day	6	0.61	.72
Treatment × Day	6	41.26	**<.001**
Population × Treatment × Day	6	0.88	.51

Of the crickets in which larvae did develop, the majority harbored two larvae that successfully emerged (Mangaia: *n* = 74 (67%); Kauai: *n* = 51 (70%)). The remaining infested crickets harbored only one larva. Populations did not significantly differ with respect to whether one or two lava(e) emerged (*χ^2^* = 0.15, *p* = .70).

**FIGURE 2:**
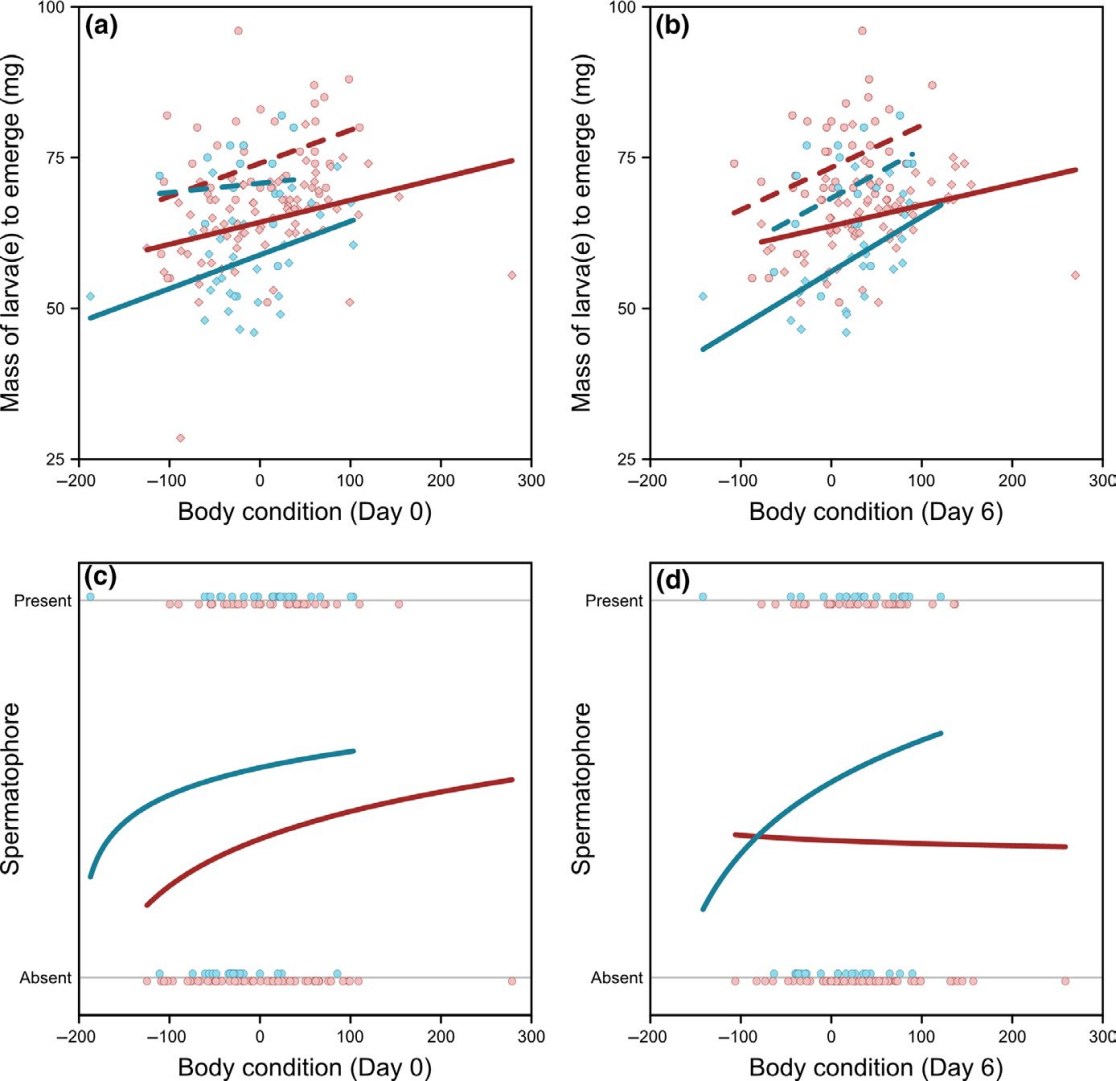
Mass of *Ormia ochracea* larvae at emergence increases with host body condition at the time of infestation (a) and six days later (b). Likelihood of producing a fresh spermatophore six days after infestation was dependent on both population of origin and male body condition prior to infestation (c), but not body condition the day that spermatophore production was assessed (d). *Upper panels*. Circle—1 larva; diamond—average mass of 2 larvae. Dashed lines show best‐fit linear regressions when only 1 larva emerged; solid lines show best‐fit linear regressions when 2 larvae emerged. *Lower panels*. Solid lines show best‐fit logistic regressions for each population. In all panels, red refers to Mangaia and blue refers to Kauai

Individuals that produced a spermatophore six days following infestation not surprisingly were also more likely to retain their testes at the time of death (*χ^2^* = 10.22, *p* = .001). Whether population of origin, number of larvae to emerge, or body condition predicted the likelihood that a male was found with a spermatophore six days into the infestation varied depending on the day on which body condition was measured. Kauai males and males with greater body condition in general were more likely than Mangaia males and males in poorer body condition to produce a spermatophore (population: *χ* ^2^ = 6.15, *p* < .01; body condition: *χ* ^2^ = 4.91, *p* = .03; number of larvae and all interaction terms: *p* > .1) when body condition was measured preinfestation (Figure 2c). However, when body condition was measured on six days following infestation, only the number of larvae to emerge was a significant predictor of spermatophore production (Figure 2d; number of larvae: *χ* ^2^ = 4.29, *p* = .04; population, body condition, and all interaction terms: *p* < .07), such that crickets with only one larva were more likely to produce a spermatophore than those that contained two larvae. Therefore, the ability to continue to produce a spermatophore in the later stages of parasitoid infestation was more strongly associated with condition at the time of infestation than at the time of spermatophore production. This is particularly interesting because all of the mass preinfestation corresponds to cricket tissue, while six days later, much of the mass is larva(e). Thus, the continued ability to produce a spermatophore is dependent on initial host cricket body condition, rather than larval size. Models examining these same factors in relation to testis retention showed no significant effects of population of origin, number of larvae, body condition (pre‐ or postinfestation), or any interactions (all *p* > .1).

**FIGURE 3:**
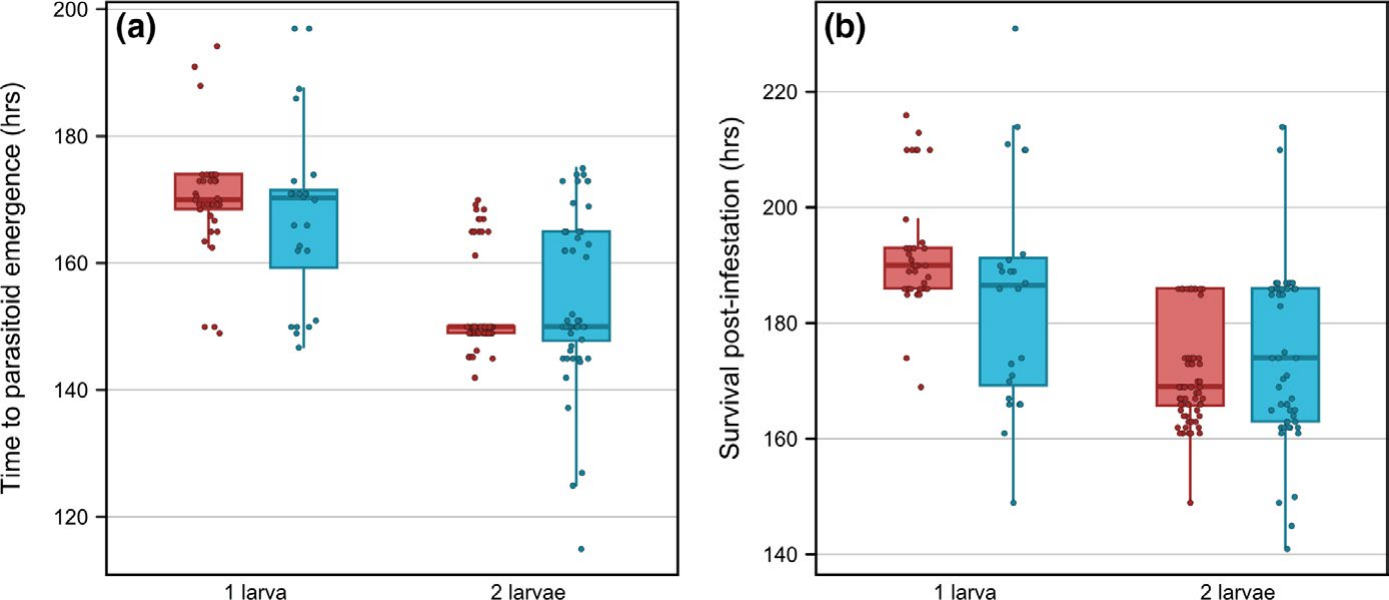
Regardless of population of origin, an increase in the number of larvae to emerge decreased the amount of time before the larvae emerged from host crickets and host survival time postinfestation (red—Mangaia; blue—Kauai)

Survival analysis showed that host crickets with one larva survived significantly longer than host crickets with two larvae (*χ* ^2^ = 16.70, *p* < .001, *hr* = 0.35); survival did not differ with respect to population of origin (*χ* ^2^ = 0.50, *p* = .48, *hr* = 1.19). Fully factorial general linear models examining whether population of origin, number of larvae to emerge, or body condition preinfestation affected time to larval emergence and host survival postinfestation found that only the number of larvae was a significant predictor of these lifespan measurements (Figure 3; Table S3). Specifically, crickets with one larva had a significantly longer time to larval emergence (*F* = 69.36, *p* < .001; all other *p* > .1) and overall longer survival (*F* = 49.16, *p* < .001; all other *p* > .1). When late stage infestation body condition (6 days) was included in the models instead of that from preinfestation, again only the number of larvae to emerge was a significant predictor of the time to larval emergence (Figure 3a; *F* = 69.79, *p* < .001; all other *p* > .1). However, the number of larvae (*F* = 43.60, *p* < .001), host body condition (*F* = 8.00, *p* = .005), and the interactions between the number of larvae and population of origin (*F* = 5.96, *p* = .02), and between body condition and population of origin (*F* = 4.13, *p* = .04), were all significant predictors of total survival time postinfestation (Table S4). Specifically, survival increased with late infestation stage body condition for crickets from Kauai, but decreased with body condition for Mangaia crickets (Figure S1).

### 3.3 Host gene expression changes in response to infestation

If the Kauai population has evolved tolerance in response to parasitoid presence, then that response may be apparent at the level of gene expression. By analyzing gene expression data, we circumvented problems associated with defining tolerance‐enabling traits at the level of organismal phenotype that are engaged during active infestation. Instead, we used a bottom‐up approach to identify suites of genes—modular gene coexpression networks—whose expression changes in coordinated fashion in response to infestation during early and late stages of infestation, and test their patterns of change with respect to known involvement in immunity, stress response, and other functional attributes. Specifically, we predicted that crickets from Kauai would show a reduced magnitude of differential regulation of immune genes and general stress response pathways, in contrast to the Mangaia population. The rationale is that the characteristic immune responses of insects to nonmobile parasitoids, for example, phenoloxidase and components of the hemocytosis and melanization pathways that lead to asphyxiation of parasitoid eggs, are not efficacious when the endoparasitoid is mobile and can evade such responses. Selection should therefore favor less transcriptional responsiveness of genes known to be involved in such *resistance* responses, and instead favor differential regulation of genes that promote *tolerance* responses.

**FIGURE 4:**
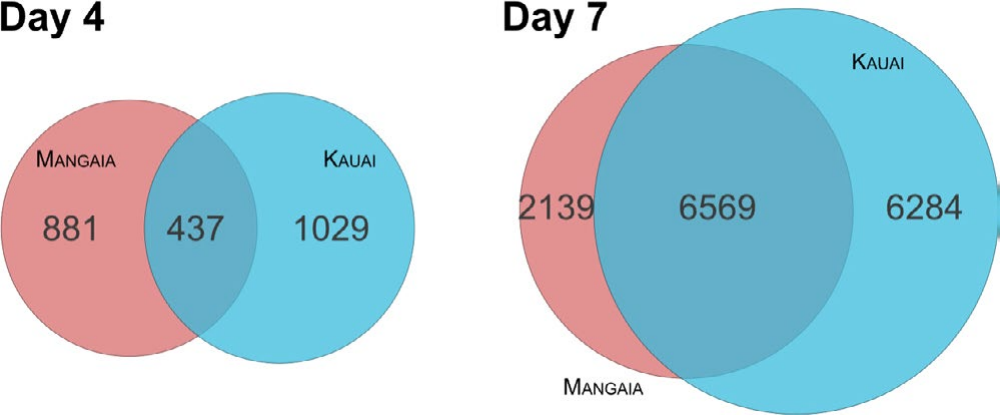
Venn diagram showing the total number of genes differentially regulated either 4 days (left) or 7 days (right) after infestation by parasitoid larvae, relative to uninfected. Genes with significant changes in expression (FDR < 0.05) in either direction relative to uninfected crickets from either the Mangaia (red) or Kauai (blue) population are included

Four days after infestation, 1318 genes were differentially expressed (DE) in infested Mangaia crickets compared with Mangaia controls (FDR < 0.05; Figure S1). A relatively similar number of genes, 1466, were DE in infested Kauai crickets compared with Kauai controls after 4 days. Most of the DE genes were unique to each population: Of the two groups of DE genes, only 437 genes were DE in both populations (Figure 4). All but two genes in this overlapping set changed in the same direction. We further tested for population‐by‐infestation interactions, which identified 113 genes that showed population‐specific expression responses to infestation (FDR < 0.05).

As predicted, the host gene expression response was greatly magnified seven days after infestation. In Mangaia and Kauai crickets, respectively, 8708 genes and 12853 genes were DE (Figure S1). Of these, 6569 were common between both populations (Figure 4), and 98.7% of the shared genes changed in the same direction (6483 genes). At this later stage of infection, 1711 genes showed population‐specific expression responses to infestation (population‐by‐infestation interaction FDR < 0.05).

### 3.4 Immune and stress response genes were not downregulated

Tolerance is typified by a lack of response to a parasite by the host, which is expected to result in a lack of changes in the transcriptional regulation of immune and stress response pathways. Specifically, we predicted that such genes would not be differentially expressed in response to parasitoid infestation in the Kauai population, but that the naïve population of Mangaia would continue to differentially regulate such genes owing to a lack of historical exposure to *O. ochracea* and the selection pressure it would impose. Our results opposed these predictions.

**FIGURE 5:**
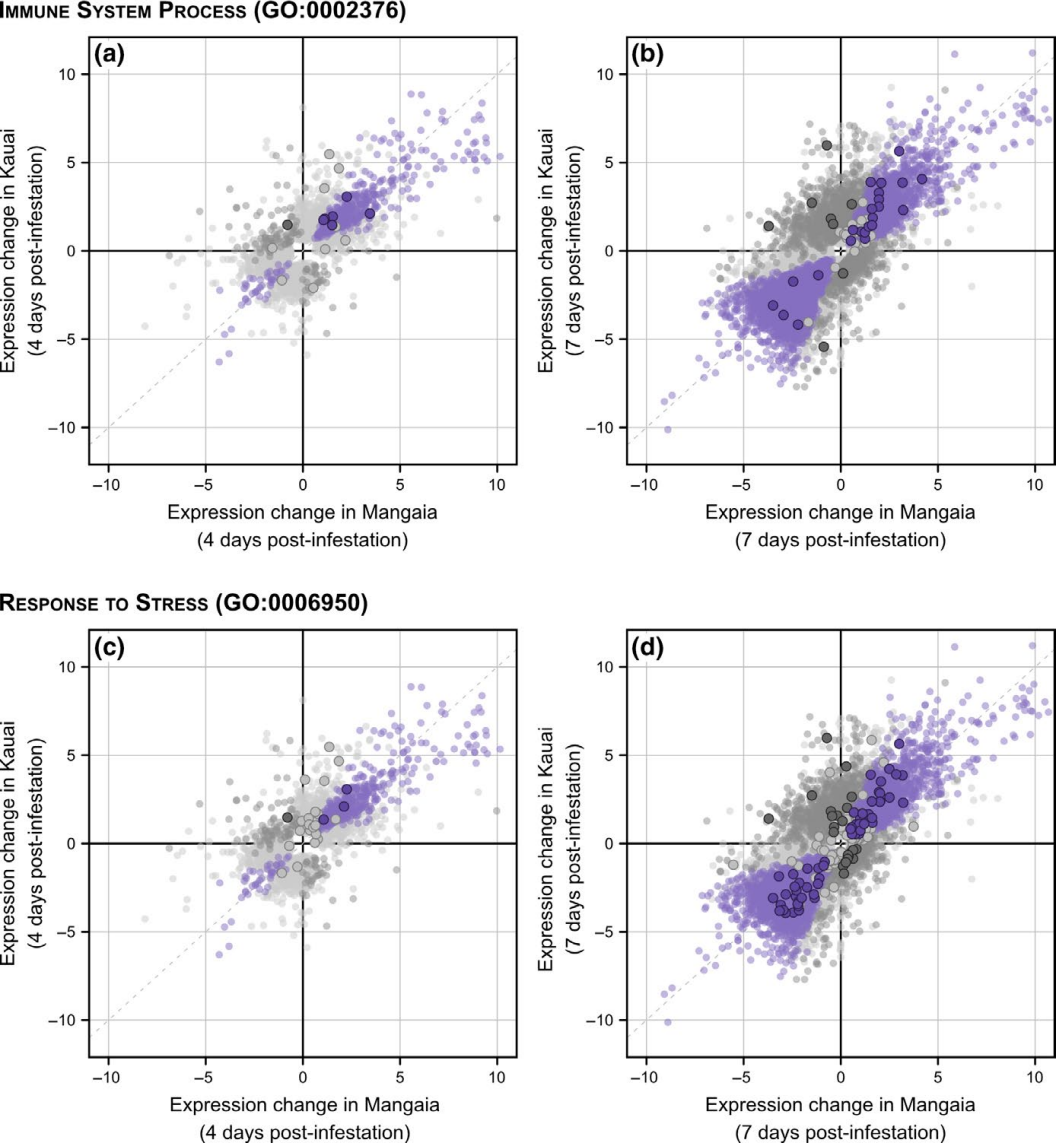
Immune and stress response genes respond similar to parasitoid infestation. Points show the log_2_ fold change in expression between the control treatment and either 4 days (a, c) or 7 days (b, d) postinfestation for each population. Only genes with significant changes in expression (FDR < 0.05) are shown in each panel. Purple indicates differentially expressed genes that respond similarly in both the Mangaia and Kauai populations. Genes in dark gray respond differently to infestation in the two populations (population‐by‐infestation interaction). Light gray points indicate genes that are significantly differentially expressed in only one population. Genes highlighted with a solid border were annotated as either immune system genes (a–b) or response to stress (c–d)

We identified 67 genes in our expression dataset that either had significant homology to phenoloxidase (a key component of insect immunity) or were annotated with the biological process ontology “immune system process.” Similarly, we identified a total of 309 genes that were annotated with the biological process term “response to stress.” Overall, infested crickets from Kauai showed more uniquely DE genes related to immunity and stress responses than those from Mangaia. Of the 67 immune genes we identified, 16 were significantly differentially regulated in at least one population 4 days after infestation by the parasitoid larvae (Figure 5A, Table S5). Six of these genes showed similar responses in both populations. Of the remainder, 6 genes were differentially expressed in Kauai but not Mangaia, while 4 genes showed the opposite pattern. After 7 days, we observed 51 immune‐related genes with significant changes in expression in at least one population (Figure 5B, Table S5). 23 of these genes responded similarly in both populations. In contrast to the early response, after 7 days there were an additional 23 immune genes DE only in the Kauai population, while only five genes were uniquely DE in crickets from Mangaia.

Genes annotated as having functions related to stress response also tend to be DE in infested crickets from Kauai. Four days after infestation, 24 stress response genes were DE in at least one population (Figure 5C). 16 of these were only DE in Kauai crickets, while 3 were DE in both populations. Only five genes were DE in Mangaia but not in Kauai. After 7 days of infestation, 180 stress‐related genes were DE in at least one population (Figure 5D), of which 57 were common to both populations, and 85 were unique to Kauai. In contrast, only 38 of these genes were DE solely in Mangaia crickets.

### 3.5 Divergence of transcriptional response to infestation between populations

Gene expression appears to be highly plastic in response to infestation in both populations, and a considerable fraction of the response is shared in both Mangaia and Kauai (Figure 5). However, there is evidence for substantial differences in the responses between the two populations as well. As described above, we observed significant interactions for many genes, especially 7 days postinfestation, as well as many genes that were significantly DE in only one population. To further investigate patterns of transcriptional divergence, we used nonmetric multidimensional scaling (nMDS), an unsupervised ordination method, to visualize differences in the global expression patterns among treatment groups.

**FIGURE 6:**
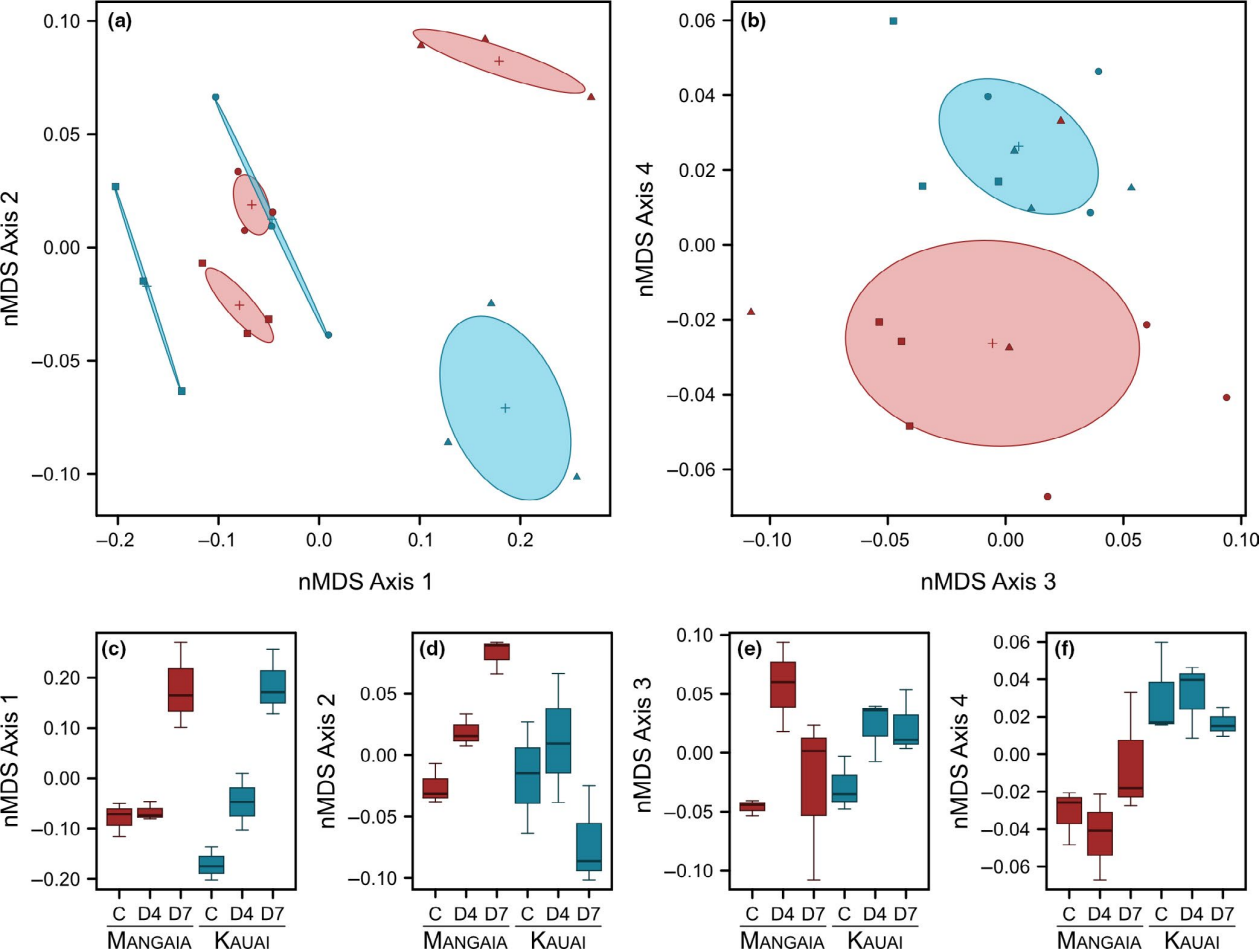
Nonmetric multidimensional scaling of transcriptional data shows divergence in expression profiles due to infestation and population origin. (a) Crickets from Mangaia (red) and Kauai (blue) exhibit distinct transcriptional profiles that diverge over the course of fly infestation on ordination axes 1 and 2. (b) The expression profiles of the Mangaia (red) and Kauai (blue) populations differ across all time points on Axis 4. (c–f) Boxplots show the divergence among treatment groups on each of the 4 axes in a nonmetric multidimensional scaling ordination (C, control; D4, 4 days postinfestation; D7, 7 days postinfestation)

Overall, we observed large differences in global expression patterns attributable to the time since infestation, which are apparent on the first ordination axis (Figure 6). We used a permutational analysis of variance (PERMANOVA) on the dissimilarity matrix to confirm that time since infestation had a highly significant effect on global patterns of gene expression (*F*_2,12_ = 10.73, *p* = .001). However, these responses diverge between the two populations on the second axis, especially 7 days after infestation, reflected by a significant population‐by‐infestation effect (PERMANOVA: *F*_2,12_ = 3.64, *p* = .002). Interestingly, nMDS axis 4 appears to indicate a subset of genes with differences in expression that separates the two populations, regardless of infestation status.

### 3.6 Coexpressed modules are associated with infestation treatment

To further clarify how transcriptional responses differ among populations, we performed a weighted gene coexpression network analysis to identify modules, which are sets of genes with highly correlated patterns of expression across samples. We identified 17 well‐supported coexpression modules, ranging in size from 32 to 4,231 genes (Table S6). An additional 9,575 genes could not be consistently assigned to any single module, and were thus designated as “Unassigned.” For each module, we calculated the eigengene, which is defined as the first principal component of the expression values for all genes in that module. After excluding the “Unassigned” group of genes, the eigengene of each of the assigned modules explained no less than 70.5% of the variance in expression, indicating that the eigengene is representative of the expression of the module’s component genes.

**FIGURE 7:**
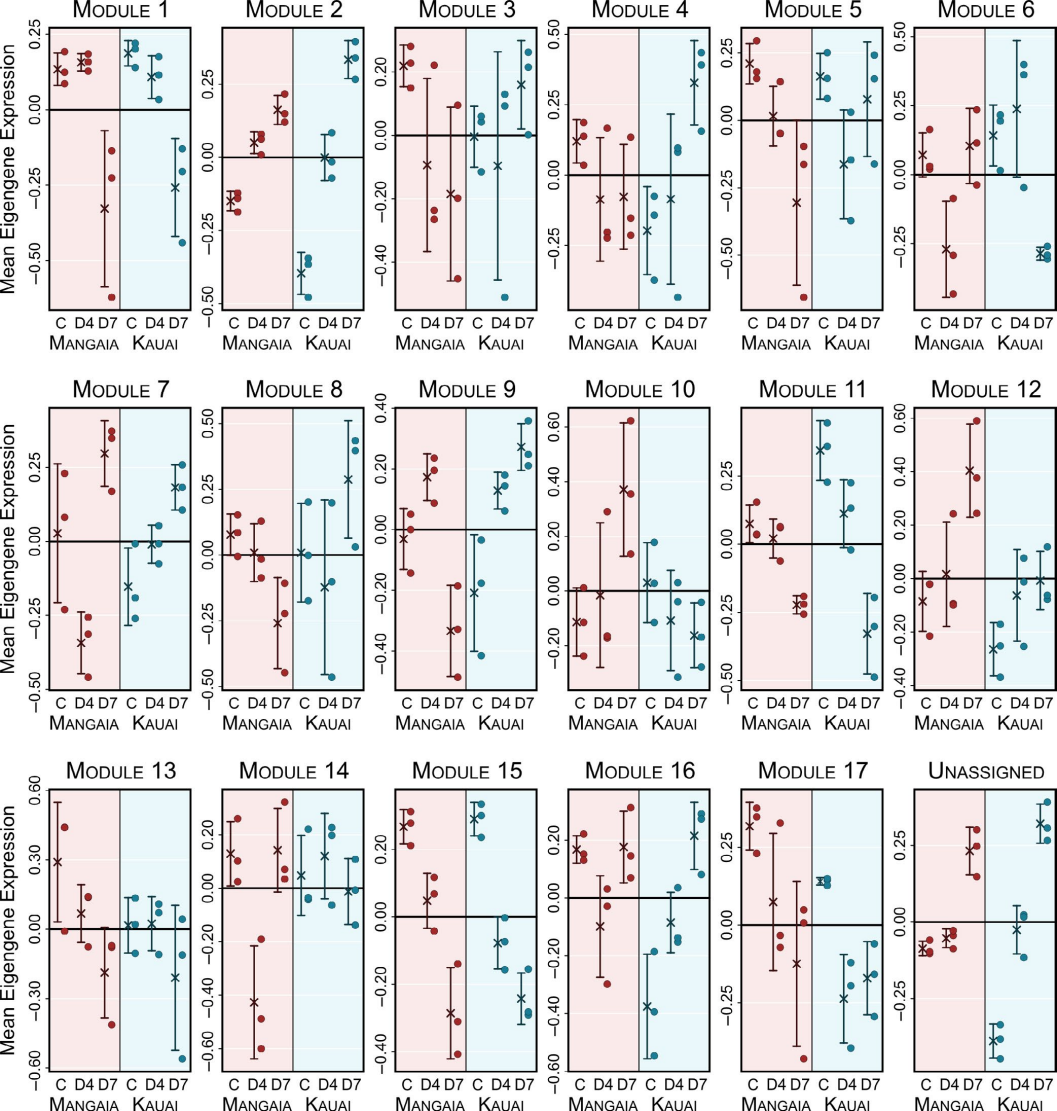
Eigengene expression in most coexpression modules is associated with time since infestation or population. Mean eigengene expression (±1 *SD*) is shown for each treatment group. Eigengene scores for each sample (circles) are also shown

As observed above, time since infestation is the factor most strongly associated with transcriptional regulation in most modules (Table S6). Eigengene expression of 12 modules is significantly affected by the time since infestation. Five modules consisted of a total of 4445 genes—Modules 1, 11, 13, 15, and 17—are primarily downregulated over the course of the infestation in crickets from both populations (Figure 7). Module 1 is a large module containing 4231 coexpressed genes, which exhibited significant enrichment of many biological processes, including cytoskeleton organization, cell cycle, and homeostatic processes, among others (Tables S6 and S7 (https://doi.org/10.17605/OSF.IO/GSMA5)). Genes involved in carbohydrate metabolism were enriched in Modules 13, 15, and 17, while those involved in DNA metabolic processes were observed in Module 11.

Module 2 and Module 12, in contrast, were upregulated in response to infestation in both cricket populations (Figure 7; Table S6). The smaller of these, Module 12, consists of 72 genes enriched for ribonucleoprotein complex assembly and translation processes. Module 2 (3773 genes) was enriched for genes relating to signal transduction and cell morphogenesis, among other processes. Many of the genes annotated as immune system process were assigned to Module 2 (22 genes; Tables S5 and S6), although this did not represent significant enrichment of immune genes within the module (Fisher’s test; *p* = .090).

To probe population‐specific responses to fly infestation, we also identified 10 modules for which the eigengene’s response to infestation differed between populations (population‐by‐infestation interaction; Table S6). These modules can be divided into four general categories based on their expression patterns. I. The first group, including Modules 2 and 11, exhibited expression changes in the same direction over time in both the Kauai and Mangaia populations, but with responses in Kauai of much greater magnitude than in Mangaia (Figure 7).

II. Module 4, Module 10 and Module 14 were comprised of genes that responded to infestation in only one population. Genes in Module 4 (429 total) tended to be upregulated 7 days after infestation, only in Kauai. This module was enriched for several functional categories including cell differentiation, cell junction processes, and nervous system processes. Similarly, the genes in Module 10 (80 genes) and Module 14 (61 genes) were differentially regulated only in Mangaia, at 7 and 4 days postinfestation, respectively. Module 10 included many genes involved in translation, while Module 14 includes components of signal transduction pathways and genes that regulate homeostasis.

III. Module 8 showed opposing responses in each population. Genes in this module tended to be downregulated 7 days postinfestation in Mangaia, but upregulated in the same time period in Kauai crickets. This module was enriched for carbohydrate metabolism and cell adhesion.

IV. Finally, Modules 6, 7, 9, and 16 all demonstrate variable responses that differ between populations, particularly in the timing of the transcriptional response to infestation. For example, Module 6 was downregulated 4 days after infestation in the Mangaia population, but the response was delayed until 7 days postinfestation in the Kauai crickets. This module was enriched for genes that generate precursor metabolites and energy. Similarly, the other modules in this group—7, 9, and 16—responded rapidly (4 days postinfestation), before returning to approximately baseline levels of expression after day 7 in the Mangaia population. In the Kauai population, these modules respond more modestly after four days, but continue to increase expression through the end of the experiment. These modules were enriched for genes involved with protein modification (Module 7), mRNA processing (Module 9), and nitrogen compound metabolism (Module 16).

## 4 DISCUSSION

Given the high likelihood of host death following successful invasion by *O. ochracea* larva(e), we hypothesized that hosts evolving in an environment where they are susceptible to such infestation are under selection to have increased tolerance but not necessarily increased immunological resistance to such parasitoids. By comparing survival and fitness measures of infested *T. oceanicus* field crickets from an island population where *O. ochracea* is completely absent to those from an island population where the parasitoid has been present for at least three decades (Zuk et al., 1993), we tested for postinfestation host differences indicative of increased survival and a tolerant strategy. We were particularly interested in population differences in reaction norms relating host fitness and survival to the number of emerging larva(e). We found no such differences between the two populations, demonstrating that tolerance to increasing severity of parasite load (i.e., more larvae) does not qualitatively differ between these two populations of *T. oceanicus*.

In general, the number of larva(e) to emerge and host body condition repeatedly emerged as important determinants of fitness and survival measures, including spermatophore production, time to first larval emergence, total survival postinfestation, and mean larval mass at emergence (a measure of parasitoid, rather than host, fitness). We did, however, find some evidence for population differences in fitness (spermatophore production) and survival (total survival time postinfestation), although in both cases significant population effects (a) were not associated with the slope of the response to different numbers of larvae and (b) only emerged from models containing either preinfestation or late stage infestation body but not at both time points. Specifically, Kauai crickets were more likely to continue to produce a spermatophore throughout the infestation when their body condition was relatively high prior to infestation. Survival, on the other hand, was dependent on late stage body condition, such that crickets from the Kauai population survived infestation for longer when they had a higher body condition. That the Mangaia population showed decreased survival with increased body condition is curious and should be explored further.

Infestation by *O. ochracea* strongly impacted gene expression profiles of their hosts, and varied over the time course of the infestation (Figure 5). Although a recent study found a relatively low level of pairwise genetic differentiation between *T. oceanicus* from one of the Hawaiian Islands (Hawaii) and Mangaia (Fst = 0.094; Pascoal et al., 2016), each population showed largely unique expression responses but with a small, core set of genes that tended to respond in the same direction (Figure 5). This divergence in the populations’ responses is most evident in the eigengene expression of 10 coexpressed modules with significant population‐by‐infestation interaction effects.

However, the patterns of differential expression that we observed here are inconsistent with mechanisms of tolerance acting through gene regulation. The functional classes of genes that we expect to show reduced plasticity in Kauai—immune system process and response to stress—tend to be equally responsive in both populations, or are more responsive in Kauai. Many of the genes known to have functions related to the immune system were assigned to Module 2. Although this does not constitute a significant enrichment of immune genes, Module 2 is nevertheless the best candidate for comprising, at least in part, the immune response to infestation in *Teleogryllus*. As a whole, the genes in this module tend to have a reduced basal level of expression in uninfested crickets from the coevolved Kauai population. However, upon infestation by parasitoid larvae, the expression of Module 2 increases significantly more in the Kauai population, as evidenced by a significant population‐by‐infestation interaction. Such a response is contrary to the predicted outcome of selection for tolerance.

The results of this study seem to effectively rule out a role for tolerance to *O. ochracea* larvae in the evolution of *T. oceanicus* populations in Hawaii. However, it is still unclear whether the changes in transcriptional regulation observed in the Kauai population represent adaptive changes in immunity and resistance to being parasitized by *O. ochracea*, or whether some changes might in fact be induced by the parasitoids themselves, which have coevolved with Kauai crickets and may therefore have acquired counter‐defenses. Although flatwing males do not seem to differ from their normal‐wing counterparts in our measures of immunity (Bailey et al., 2011, this study), a recent study found that flatwing males produce significantly more offspring per individual mating than do normal‐wing males (Heinen Kay et al., 2019). Traits such as fitness and immunity undoubtedly affect transcription of large numbers of genes relating to wing and/or testis development, among other functions (Rayner et al., 2019). In addition, a significant limitation of using crickets derived from extremely isolated wild populations is that we cannot account for all of the myriad morphological, behavioral, or physiological traits that similarly may have diverged between populations, and which may not be directly related to co‐occurrence with the parasitoid fly. Future studies therefore would benefit from the inclusion of additional *T. oceanicus* populations that do (e.g., populations on additional Hawaiian Islands) and do not (e.g., populations throughout the South Pacific and Australia) share a history of coevolution with *O. ochracea*. Such population‐level replication would provide greater support that the differences seen here are in fact due to parasitoid‐associated selection.

Finally, we speculate that the lack of evidence for tolerance in this study may be related to the strength of selection acting concurrently on mechanisms conferring resistance and tolerance in this system. Why should different populations of the same species produce different transcriptional responses to invasion by a parasitoid? The fact that Kauai crickets have coevolved with the parasitoid for many generations would logically contribute to this difference, but it is evident that that coevolution has not given Kauai hosts an upper hand: Fitness is similarly, and dramatically, decreased in this population compared with a parasitoid‐naïve population. Could it be that the parasitoid itself is locked into an arms race and has evolved additional larval strategies to thrive within Kauai hosts? These could involve parasitoid‐directed changes in host gene expression; evolutionary models of such processes predict the accumulation of multiple defenses, counter‐defenses, and generally accelerated evolution. This could account for the larger magnitude and extent of differential gene expression in Kauai crickets, alongside similar fitness outcomes of parasitization.

## CONFLICT OF INTEREST

None declared.

## AUTHOR CONTRIBUTIONS

SLB: Study conceptualization; infestations ; analyses of survival and fitness. KS: Gene expression analyses. MZ: Cricket collection. All authors contributed to the interpretation, writing, and editing of the manuscript.

## Supporting information

Fig S1

Table S1‐6

## Data Availability

Sequence data for this study have been deposited in the NCBI Gene Expression Omnibus (GEO) database as part of series GSE15139 with accession numbers GSM4584950‐4584967. Raw data from pertaining to survival and fitness, and Supplemental Table S7 containing details of module GOs are archived at https://doi.org/10.17605/OSF.IO/GSMA5.
