# Supplementary material for: Immunogenetic and tolerance strategies against a novel parasitoid of wild field crickets
Source: Ecol Evol. 2020 Oct 26;10(23):13312–26. doi: 10.1002/ece3.6930 (PMC7713935; doi:10.1002/ece3.6930)

Figure S1. Survival increases with body condition 6 days following infestation for *T. oceanicus* from Kauai (blue circles), but decreases with body condition at the same timepoint for *T. oceanicus* from Mangaia.

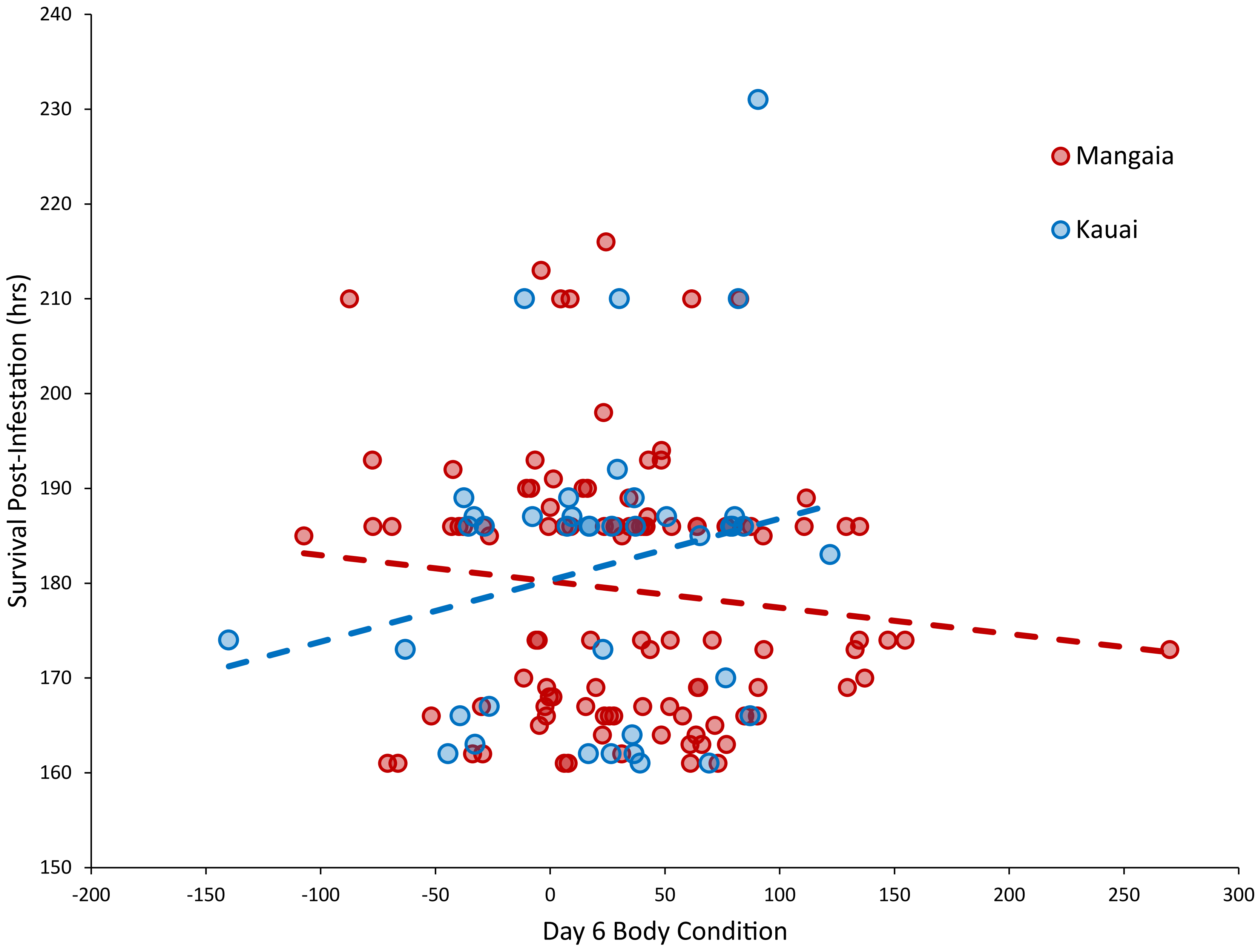

Supplement: Supplementary file 1 — Fig S1 [file ECE3-10-13312-s001.pdf]
