# Supplementary material for: Immunogenetic and tolerance strategies against a novel parasitoid of wild field crickets
Source: Ecol Evol. 2020 Oct 26;10(23):13312–26. doi: 10.1002/ece3.6930 (PMC7713935; doi:10.1002/ece3.6930)
Supplement: Supplementary file 2 — Table S1‐6 [file ECE3-10-13312-s002.docx]

**Table S1** Results of repeated measures ANOVA least squares *post hoc* effect slices examining the significant interaction effects of day of infestation (D0-6) and treatment on host body condition. In all cases where the day is significant, infested crickets had higher body condition than controls. Complete model also examined wing morph, thus only Kauai crickets are included in this analysis. Only effect slices of factors found significant in the full model are shown.

**Body condition**

df *F p*

Day 0 1 1.69 0.19

Day 1 1 0.21 0.65

Day 2 1 0.07 0.79

Day 3 1 3.42 0.07

Day 4 1 10.41 **0.001**

Day 5 1 11.20 **<0.001**

Day 6 1 10.84 **<0.001**

**Table S2** Results of repeated measures ANOVA least squares *post hoc* effect slices examining the significant interaction effects of population (flatwing or normal) and the interaction between day of infestation (D0-6) and treatment on host body condition. In all cases where the day is significant, infested crickets had higher body condition than controls. Only effect slices of factors found significant in the full model are shown.

**Body condition**

df *F p*

Mangaia x Treatment 1 37.25 **<0.001**

Kauai x Treatment 1 0.84 0.36

Day 0 x Treatment 1 1.19 0.28

Day 1 x Treatment 1 0.69 0.41

Day 2 x Treatment 1 1.84 0.17

Day 3 x Treatment 1 12.64 **<0.001**

Day 4 x Treatment 1 37.43 **<0.001**

Day 5 x Treatment 1 54.37 **<0.001**

Day 6 x Treatment 1 62.48 **<0.001**

**Table S3** Results of fully factorial least squares effects tests examining the effects of the number of larvae to emerge, population of origin, and host body condition at the start of the experiment on the mean mass of larvae to emerge.

**Mean larval mass Time to emergence Survival post-infestation**

*F p F p F p*

Larvae 56.26 **<0.001** 69.36 **<0.001** 49.16 **<0.001**

Population 5.29 **0.02** 3.43 0.07 1.32 0.25

Body condition 9.27 **0.003** 2.51 0.11 1.73 0.19

Larvae x Population 1.43 0.23 0.53 0.47 2.67 0.10

Larvae x Body condition 0.01 0.94 0.09 0.77 0.00 0.96

Population x Body condition 0.01 0.91 1.40 0.24 0.24 0.62

Larvae x Population x Body condition 0.26 0.61 0.64 0.42 0.09 0.76

**Table S4** Results of fully factorial least squares effects tests examining the effects of the number of larvae to emerge, population of origin, and host body condition six days after infestation on the mean mass of larvae to emerge.

**Mean larval mass Time to emergence Survival post-infestation**

*F p F p F p*

Larvae 62.30 **<0.001** 70.14 **<0.001** 43.43 **<0.001**

Population 17.04 **<0.001** 1.65 0.20 0.00 1.00

Body condition 29.42 **<0.001** 2.09 0.15 7.77 **0.006**

Larvae x Population 1.38 0.24 1.62 0.21 5.82 **0.02**

Larvae x Body condition 0.57 0.45 0.16 0.69 1.40 0.24

Population x Body condition 2.39 0.12 0.70 0.40 4.27 **0.04**

Larvae x Population x Body condition 0.32 0.57 0.02 0.88 3.26 0.07

**Table S5.** Gene expression change in response to infestation for subset of genes annotated with immune system functions.

|  |  |  | **4 days post-infestation (log2 fold change)** | | **7 days post-infestation (log2 fold change)** | |
| --- | --- | --- | --- | --- | --- | --- |
| **Transcript ID (Gene ID)** | **Gene Description** | **Module** | **Mangaia** | **Kauai** | **Mangaia** | **Kauai** |
| MSTRG.7687 | Toll-like receptor Tollo | 2 | 1.85 | **4.67 *** | **3.00 *** | **5.64 *** |
| MSTRG.7273 (TOG005518) | phenoloxidase 2-like | 2 | **3.45 *** | **2.12 *** | **4.18 *** | **4.07 *** |
| MSTRG.22165 | toll-like receptor 2 | NA | 1.36 | **5.47 *** | -0.72 | **5.97 *** |
| MSTRG.7205 | Toll-like receptor Tollo | 2 | **2.26 *** | **3.07 *** | **3.18 *** | **3.86 *** |
| MSTRG.872 | TLR4 interactor with leucine rich repeats | Un | 1.10 | **3.54 *** | **1.54 *** | **3.89 *** |
| MSTRG.279 (TOG000204) | adenosine deaminase | 1 | 0.54 | **-2.09 *** | -0.88 | **-5.43 *** |
| MSTRG.13096 (TOG010460) | hypothetical protein C0J52_16712 | 2 | **2.19 *** | 0.60 | **2.08 *** | **3.85 *** |
| MSTRG.10990 (TOG008652) | phenoloxidase 2-like | 2 | **1.11 *** | **1.82 *** | **1.96 *** | **3.29 *** |
| MSTRG.6375 (TOG004887) | interleukin-1 receptor-associated kinase 1-binding protein 1 | 1 | -0.27 | -1.15 | **-2.95 *** | **-3.63 *** |
| MSTRG.21787 | adhesion G protein-coupled receptor A3 | Un | -2.41 | 0.42 | **-3.71 *** | 1.41 |
| MSTRG.13095 | hypothetical protein C0J52_03247 | 2 | **1.69 *** | 1.37 | **1.96 *** | **2.91 *** |
| MSTRG.23133 | Cytochrome b5 | 1 | 0.30 | -1.03 | **-2.20 *** | **-4.18 *** |
| TOG006794 | toll-like receptor 13 | NA | 1.22 | 0.94 | **3.21 *** | **2.31 *** |
| MSTRG.11188 (TOG008811) | phenoloxidase 2-like | NA | 0.04 | -1.56 | -1.67 | **-4.04 *** |
| MSTRG.17307 | PPO | 2 | **1.06 *** | **1.75 *** | **1.96 *** | **2.50 *** |
| MSTRG.9040 (TOG007013) | peptidoglycan-recognition protein SC2 | 15 | -1.08 | **-1.67 *** | **-2.45 *** | **-1.74 *** |
| MSTRG.2949 (TOG002337) | aquaporin-4 isoform X2 | 1 | 0.20 | 0.07 | **-3.48 *** | **-3.09 *** |
| MSTRG.22446 (TOG017988) | toll-like receptor 2 | 2 | 0.90 | 1.38 | **1.60 *** | **2.38 *** |
| MSTRG.23335 | AGAP011476-PA-like protein | 4 | -0.79 | 1.03 | -1.49 | **2.72 *** |
| MSTRG.10135 (TOG007875) | Phenoloxidase subunit A3 | Un | **1.53 *** | **1.94 *** | 1.04 | **1.50 *** |
| MSTRG.1405 (TOG001092) |  | 2 | 0.87 | 0.54 | 1.13 | **2.75 *** |
| MSTRG.10136 | PPO | Un | **1.51 *** | **1.46 *** | **1.24 *** | **1.05 *** |
| MSTRG.21709 (TOG017379) | DChain D, Coupling Of Remote Alternating-access Transport Mechanisms For Protons And Substrates In The Multidrug Efflux Pump Acrb | Un | 1.01 | 0.41 | **1.64 *** | **1.87 *** |
| MSTRG.9480 (TOG007332) | Toll-like receptor 13 | Un | -0.80 | **1.48 *** | -0.51 | **1.82 *** |
| MSTRG.22070 (TOG017693) | peptidoglycan-recognition protein 2-like | 2 | 0.67 | 0.74 | **1.61 *** | **1.45 *** |
| TOG013608 | CD180 antigen | NA | 0.48 | 0.89 | 1.06 | **1.72 *** |
| MSTRG.11402 (TOG008988) | hypothetical protein C0J52_03247 | NA | -0.08 | 0.93 | 1.34 | **1.77 *** |
| MSTRG.17457 (TOG013962) | protein eiger | 2 | 0.52 | 0.70 | **1.44 *** | **1.37 *** |
| MSTRG.5414 | toll-like receptor 6 | 2 | -0.10 | 0.67 | 0.57 | **2.64 *** |
| MSTRG.19162 (TOG015341) | Serine hydroxymethyltransferase, mitochondrial | 2 | 0.91 | 0.52 | **1.56 *** | 0.85 |
| MSTRG.2317 (TOG001826) | Lola protein isoform A | 1 | -0.20 | -0.89 | **-1.16 *** | **-1.38 *** |
| MSTRG.10137 (TOG007876) | phenoloxidase subunit A3 | Un | 0.83 | 0.96 | 0.29 | **1.09 *** |
| TOG006979 | Phosphoacetylglucosamine mutase | Un | -0.64 | 0.52 | -0.39 | **1.52 *** |
| MSTRG.16898 (TOG013506) | Syntaxin-5 | 2 | 0.24 | 0.70 | 0.58 | **1.51 *** |
| MSTRG.5564 | leucine rich repeat protein | 2 | 0.53 | 0.55 | 0.83 | **1.06 *** |
| MSTRG.6730 | phenoloxidase-activating factor 2 isoform X1 | 2 | -0.02 | 0.76 | **1.04 *** | **1.10 *** |
| MSTRG.4830 (TOG003756) | ras-related protein Ral-a isoform X1 | 2 | 0.33 | 0.52 | 0.57 | **1.30 *** |
| MSTRG.3504 | tubulointerstitial nephritis antigen-like | 2 | 0.65 | 0.10 | **1.25 *** | **0.69 *** |
| TOG018967 | Lola protein isoform A | NA | 0.48 | 0.37 | **1.27 *** | 0.45 |
| TOG006749 | small ubiquitin-related modifier 3 | 2 | -0.14 | 0.48 | **0.63 *** | **1.19 *** |
| MSTRG.18424 (TOG014766) | Peptidoglycan-recognition protein LB | Un | -0.64 | 0.58 | 0.26 | **0.90 *** |
| MSTRG.12613 (TOG010082) | sterile alpha and TIR motif-containing protein 1 isoform X1 | Un | -0.96 | 0.28 | -0.06 | **1.07 *** |
| MSTRG.14886 (TOG011914) | Tumor suppressor candidate 2 | Un | 0.13 | 0.75 | 0.48 | **0.79 *** |
| MSTRG.17746 | tubulointerstitial nephritis antigen-like | Un | 0.16 | -0.11 | 0.67 | **1.14 *** |
| MSTRG.18399 | mitoferrin-1-like isoform X1 | Un | **1.15 *** | 0.09 | -0.39 | -0.40 |
| MSTRG.15184 (TOG012134) | DNA-directed RNA polymerase III subunit RPC8 | 6 | -0.32 | -0.23 | 0.12 | **-1.28 *** |
| MSTRG.23640 (TOG018880) | ryanodine receptor | Un | **-1.57 *** | 0.17 | -0.06 | -0.07 |
| MSTRG.18304 (TOG014671) | stimulator of interferon genes protein | 2 | -0.09 | 0.60 | 0.17 | **0.95 *** |
| MSTRG.9172 (TOG007123) | DNA mismatch repair protein Mlh1 isoform X1 | 1 | 0.42 | -0.09 | -0.29 | **-0.95 *** |
| MSTRG.18615 (TOG014890) | phenoloxidase-activating factor 2 isoform X2 | Un | 0.06 | 0.32 | **0.73 *** | 0.41 |
| MSTRG.16997 (TOG013561) | microphthalmia-associated transcription factor isoform X2 | 2 | -0.10 | 0.35 | 0.31 | **0.74 *** |
| MSTRG.21064 (TOG016846) | ADP-ribosylation factor 1 | 2 | 0.12 | 0.26 | **0.52 *** | **0.58 *** |
| MSTRG.22129 (TOG017732) | SET and MYND domain-containing protein 5 | Un | 0.30 | 0.21 | **0.71 *** | -0.03 |

**Table S6.** Gene expression module eigengene ANOVA results and summary of enriched gene ontology functions.

| **Module** | **Number of Genes** | **% Variance Explained by PC1** | **Eigengene Effects** | **F** | **df(num), df(denom)** | ***P*** |  | **Functional Enrichment** |
| --- | --- | --- | --- | --- | --- | --- | --- | --- |
| 1 | 4231 | 83.5% | Population | 0.16 | 1, 12 | 0.700 |  | cytoskeleton organization, cell motility, reproduction, cell cyle, homeostatic process, others |
|  |  |  | Infestation Stage | 22.46 | 2, 12 | **< 0.001** | ***** |  |
|  |  |  | Pop x Infest | 0.36 | 2, 12 | 0.706 |  |  |
| 2 | 3773 | 70.5% | Population | 2.30 | 1, 12 | 0.155 |  | signal transduction, vesicle-mediated transport, protein modification, cell morphogenesis, others |
|  |  |  | Infestation Stage | 120.23 | 2, 12 | **< 0.001** | * |  |
|  |  |  | Pop x Infest | 19.29 | 2, 12 | **< 0.001** | * |  |
| 3 | 492 | 78.1% | Population | 0.14 | 1, 12 | 0.720 |  | small molecule metabolic process, lipid metabolic process |
|  |  |  | Infestation Stage | 1.19 | 2, 12 | 0.336 |  |  |
|  |  |  | Pop x Infest | 2.37 | 2, 12 | 0.136 |  |  |
| 4 | 429 | 75.1% | Population | 0.10 | 1, 12 | 0.752 |  | cell differentiation, nervous system process, cell junction organization, others |
|  |  |  | Infestation Stage | 1.95 | 2, 12 | 0.185 |  |  |
|  |  |  | Pop x Infest | 5.19 | 2, 12 | **0.024** | * |  |
| 5 | 201 | 78.2% | Population | 0.37 | 1, 12 | 0.557 |  | carbohydrate metabolic process, lipid metabolic process |
|  |  |  | Infestation Stage | 4.67 | 2, 12 | **0.032** | * |  |
|  |  |  | Pop x Infest | 3.80 | 2, 12 | 0.053 |  |  |
| 6 | 198 | 78.8% | Population | 0.82 | 1, 12 | 0.382 |  | generation of precursor metabolites and energy |
|  |  |  | Infestation Stage | 2.76 | 2, 12 | 0.103 |  |  |
|  |  |  | Pop x Infest | 14.03 | 2, 12 | **0.001** | * |  |
| 7 | 108 | 77.4% | Population | 0.04 | 1, 12 | 0.841 |  | cellular protein modification process |
|  |  |  | Infestation Stage | 15.78 | 2, 12 | **< 0.001** | * |  |
|  |  |  | Pop x Infest | 6.66 | 2, 12 | **0.011** | * |  |
| 8 | 89 | 79.6% | Population | 1.48 | 1, 12 | 0.248 |  | carbohydrate metabolic process, nucleobase-containing compound catabolic process, cell adhesion |
|  |  |  | Infestation Stage | 0.39 | 2, 12 | 0.684 |  |  |
|  |  |  | Pop x Infest | 5.18 | 2, 12 | **0.024** | * |  |
| 9 | 81 | 72.7% | Population | 5.20 | 1, 12 | **0.042** | * | mRNA processing, symbiont process |
|  |  |  | Infestation Stage | 8.03 | 2, 12 | **0.006** | * |  |
|  |  |  | Pop x Infest | 18.56 | 2, 12 | **< 0.001** | * |  |
| 10 | 80 | 83.1% | Population | 3.25 | 1, 12 | 0.097 |  | translation, ribosome biogenesis |
|  |  |  | Infestation Stage | 1.37 | 2, 12 | 0.290 |  |  |
|  |  |  | Pop x Infest | 4.96 | 2, 12 | **0.027** | * |  |
| 11 | 76 | 72.1% | Population | 3.22 | 1, 12 | 0.098 |  | DNA metabolic process |
|  |  |  | Infestation Stage | 37.04 | 2, 12 | **< 0.001** | * |  |
|  |  |  | Pop x Infest | 5.24 | 2, 12 | **0.023** | * |  |
| 12 | 72 | 80.0% | Population | 10.16 | 1, 12 | **0.008** | * | translation, ribonucleoprotein complex assembly |
|  |  |  | Infestation Stage | 9.57 | 2, 12 | **0.003** | * |  |
|  |  |  | Pop x Infest | 1.97 | 2, 12 | 0.182 |  |  |
| 13 | 68 | 80.1% | Population | 1.40 | 1, 12 | 0.260 |  | carbohydrate metabolic process |
|  |  |  | Infestation Stage | 4.75 | 2, 12 | **0.030** | * |  |
|  |  |  | Pop x Infest | 0.71 | 2, 12 | 0.512 |  |  |
| 14 | 61 | 78.5% | Population | 1.97 | 1, 12 | 0.185 |  | signal transduction, homeostatic process |
|  |  |  | Infestation Stage | 4.35 | 2, 12 | **0.038** | * |  |
|  |  |  | Pop x Infest | 9.10 | 2, 12 | **0.004** | * |  |
| 15 | 38 | 74.2% | Population | 0.25 | 1, 12 | 0.625 |  | carbohydrate metabolic process |
|  |  |  | Infestation Stage | 63.61 | 2, 12 | **< 0.001** | * |  |
|  |  |  | Pop x Infest | 1.82 | 2, 12 | 0.204 |  |  |
